# Identification of novel diagnostic and prognostic miRNA signatures in endometrial cancer

**DOI:** 10.18632/genesandcancer.144

**Published:** 2017-05

**Authors:** Muralidharan Jayaraman, Rangasudhagar Radhakrishnan, Cara A. Mathews, Mingda Yan, Sanam Husain, Katherine M. Moxley, Yong Sang Song, Danny N. Dhanasekaran

**Affiliations:** ^1^ Stephenson Cancer Center, The University of Oklahoma Health Sciences Center, Oklahoma City, OK, USA; ^2^ Department of Cell Biology, The University of Oklahoma Health Sciences Center, Oklahoma City, OK, USA; ^3^ Department of Obstetrics and Gynecology, The University of Oklahoma Health Sciences Center, Oklahoma City, OK, USA; ^4^ Department of Pathology, The University of Oklahoma Health Sciences Center, Oklahoma City, OK, USA; ^5^ Department of Obstetrics and Gynecology, College of Medicine, Seoul National University, Seoul, S. Korea

**Keywords:** Endometrial cancer, miRNA, miR-142, miR-15a, therapy response

## Abstract

With the goal of identifying diagnostic and prognostic biomarkers in endometrial cancer, miRNA-profiling was carried out with formalin-fixed paraffin embedded (FFPE) tissue samples from 49 endometrial cancer patients. Results using an 84-cancer specific miRNA panel identified the upregulation of miR-141-3p and miR-96-5p along with a downregulation of miR-26, miR-126-3p, miR-23b, miR-195-5p, miR-374a and let-7 family of miRNAs in endometrial cancer. We validated the dysregulated expression of the identified miRNAs in a panel of endometrial cancer cell-lines. Immunohistochemical analysis of the tissue micro array derived from these patients established the functional correlation between the decreased expression of tumor suppressive miRNAs and their target oncogenes: ERBB2, EGFR, EPHA2, BAX, GNA12, GNA13, and JUN. Comparative analysis of the samples from the patients with extended progression-free survival (PFS) ( > 21 months) versus the patients with the PFS of < 21 months indicated increased expression of tumor suppressive miR-142-3p, miR-142-5p, and miR-15a-5p in samples from extended PFS patients. In addition to defining a specific set of miRNAs and their target genes as potential diagnostic biomarkers, our studies have identified tumor suppressive miR-142 cluster and miR-15a as predictors of favorable prognosis for therapy response in endometrial cancer.

## INTRODUCTION

Endometrial cancer is one of the major gynecological cancer that will be affecting more than 61,000 patients with the anticipated death of 10,920 patients in 2017 [1]. While early detection has significantly increased the overall survival rate, 20% of the cases have poor prognosis with a median survival rate of just an year [2]. This is primarily due to the resistance to chemotherapy and disease recurrence. No precise molecular tags are available at this time to be used as a good diagnostic or prognostic biomarker for therapy resistance and disease recurrence in endometrial cancer [3-6]. Identifying a prognostic marker will greatly benefit the patients since it can be used as a predictor for therapy response so as to target treatment to the patients who would be greatly benefited from the specific adjuvant therapy. With the goal of identifying such a prognostic marker, we focused on defining the changes in miRNA profiles that could be associated with therapy resistance in endometrial cancer patients. In this study, we investigated the changes in miRNA profiles using FFPE-samples derived from a cohort of endometrial cancer patients. Using FFPE-samples from patients who had progressive disease during or shortly following chemotherapy and patients who remained without disease recurrence, expression profiles of miRNAs were analyzed using a cancer-specific 84-miRNA analysis panel.

Our results presented here indicates that the endometrial cancer tissues showed an increase in the expression of several oncomiRs with a concomitant decrease in the expression of tumor suppressor miRNAs compared to control samples. Overexpression of known oncomiRs include miR-32-5p, miR-96-5p, miR-141-3p, miR-142-5p, and miR-210. Whereas, the tumor suppressive miRNAs that showed decreased expression in endometrial cancer tissue are: miR-26, miR-126-3p, miR-23b, miR-195-5p, miR-374a and let-7 family of miRNAs. Validation of the expression of these miRNAs in endometrial cancer cell lines HEC1A, HEC1B RL95, Ishikawa and/or HEC50 indicated a similar increased expression of the representative oncomiRs along with the downregulation of tumor suppressive miRNAs, thus validating them with the oncogenic signature of endometrial cancer. Our results also indicate that the expression of the tumor-specific miRNAs can be correlated with their effect on their respective target mRNAs indicated by an increase in the expression of several oncogenic proteins such ERBB2, EGFR, EPHA2, BAX, GNA12, GNA13, and JUN, further substantiating the validity of these miRNAs as potential tumor-specific biomarkers in endometrial cancers. Differential analysis of miRNA profile between recurrent and non-recurrent patients indicated that the upregulation of the tumor suppressor miR-142-3p, miR-142-5p, and miR-15a-5p along with a drastically increased expression of miR-96-5p was observed in the samples derived from patients who showed a PFS of more than 21 months. In contrast, the samples derived from patients with PFS of less than 21 months showed little or no expression of miR-142-3p, miR-142-5p, and miR-15a-5p and relatively weaker expression of miR-96-5p. Thus, our studies presented here identifies the potential role of miR-142-3p, miR-142-5p, and miR-15a-5p, as favorable prognostic indicators for therapy response in endometrial cancer.

## RESULTS AND DISCUSSION

### Expression profiling of miRNAs in endometrial cancer samples

**Figure 1:**
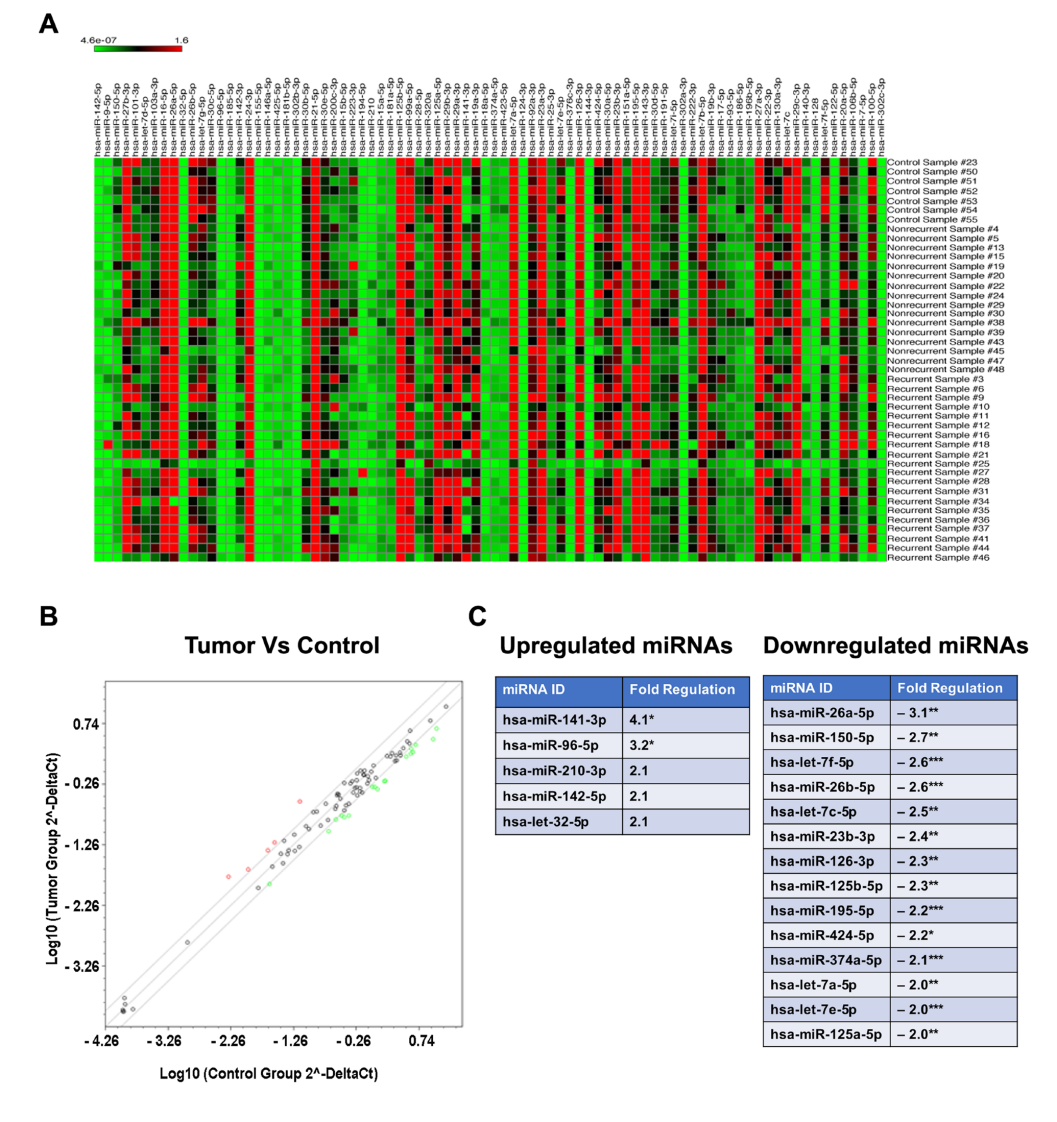
Expression analysis of miRNAs in FFPE samples from endometrial cancer patients Heat map and scatter plot images represent results from miRNA profiling using FFPE samples derived from normal or endometrial cancer patients by RT-qPCR. A. Heat map shows differential miRNA expression profiles between normal and endometrial cancer patient derived samples. Upregulated genes are shown in red and downregulated genes are in green. B. In the scatter plot, upregulated miRNAs (> 2-fold) are denoted by red circles and downregulated miRNAs (< 2-fold) are denoted by green open circles. *C*. Upregulated and downregulated miRNAs in FFPE samples from endometrial cancer tissue along with the fold changes are listed. Significant differences in the fold changes are indicated by p-values: * - <0.05; ** - <0.01; *** - <0.001.

To identify a panel of miRNAs that can serve as a diagnostic and/or prognostic marker in endometrial cancer, we carried out a retrospective cohort analysis. Expression of miRNA in endometrial cancer patients using archived FFPE samples from 49 patients who underwent surgery for advanced endometrial cancer was carried out. Based on the survival and recurrence data, the subjects were divide into two clinical groups namely, “responders to chemotherapy” and “non-responders to chemotherapy”. The responder group (n = 26) is defined by the subjects without disease recurrence for more than 21 months whereas the non-responders (n = 23) represented the subjects whose disease continued to progress either during chemotherapy (n = 6) or soon after the completion of chemotherapy (n = 17). While the responders had progression free survival with a median value of 52 months, all of the non-responders succumbed to the disease by 3 – 21 months. Based on the quality of the FFPE blocks, specimens from 16 responders and 20 non-responders were processed for miRNA profile analysis. Normal endometrial tissue from six FFPE specimens were used as the control group. The miRNA profiling was carried out by PCR methods to monitor the expression of 84 well-characterized cancer related miRNAs. As shown in Fig. 1, the samples from cancer tissues showed altered expression profile of several specific miRNAs (Figure 1A). A comparative scatter blot between control and patient tumor samples, derived from this dataset identified the unique set of miRNAs that showed altered expression in endometrial cancer (Figure 1B). Our results indicated that only miR-141-3p and miR-96-5p showed significant increase in cancer tissue samples compared to normal tissue sample by two folds. Increased expression seen with miR-96-5p, miR-142-5p, and miR-32-5p were not statistically significant. It is of interest to note here that miR-141-3p [8] and miR-96-5p have been identified as OncomiRs in many cancers [8-21]. In striking contrast, endometrial cancer tissue samples showed a significant decrease in fourteen tumor-suppressive miRNAs (Figure 1C). It is of interest to note here that all of the fourteen downregulated miRNAs, namely, miR-26a-5p [22, 23], miR-150-5p [24, 25], let-7f-5p [26, 27], miR-26b-5p [22, 23, 28], let-7c-5p [26, 27], miR-23b-5p [29-31], miR-126-3p [32], miR-125b-5p [33], hsa-miR-195-5p [34, 35], miR-424-5p [36, 37], hsa-miR-374a-5p [38-40], let-7a-5p [26, 27] , let-7e-5p [26, 27], and miR-125a-5p [41, 42] are known to have tumor-suppressive function in many cancers. Taken together with the upregulated genes, our results identify a panel of miRNAs that correlates with the tumor phenotype. Our results showing the upregulation of miR-141 miR-96, and miR-32 in endometrial cancer are in agreement with a previous report that identified the increased expression of these miRNAs in endometrial cancer [43]. Our studies presented here extend this further with a novel finding that identify an array of fourteen tumor-suppressive miRs that are downregulated in endometrial cancer.

### Validation of differentially expressed miRNAs

**Figure 2:**
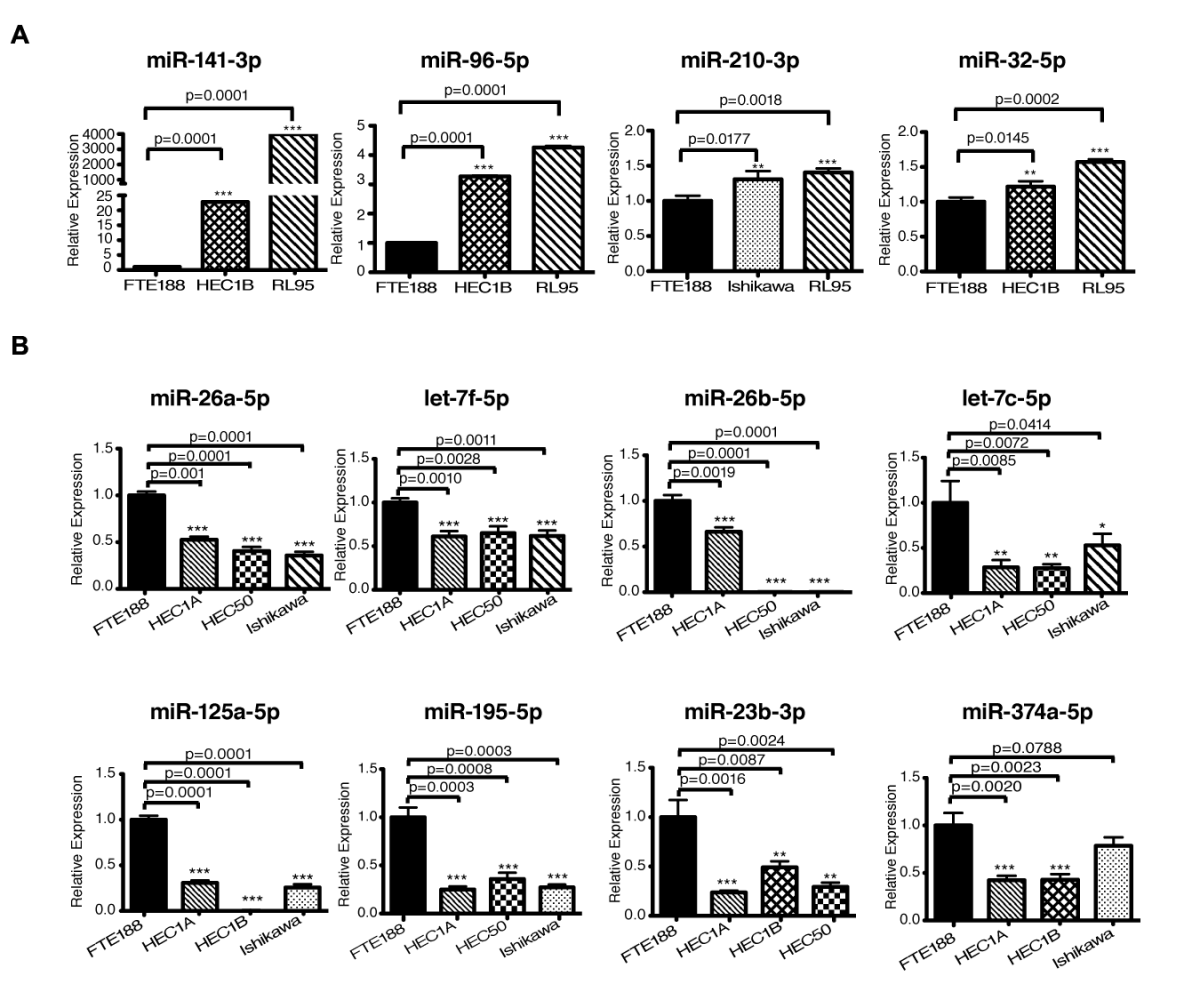
Expression analysis of miRNAs in endometrial cancer cell lines A. Expression of upregulated miRNAs (miR-32-5p, miR-96-5p, miR-141-3p, miR-142-5p, and miR-210) in HEC1B, RL95 and Ishikawa cells in comparison with the normal fallopian tube derived epithelial cells (FTE188) are presented. Values are presented as Mean ± SEM (n=3). B. Expression of downregulated miR-26a-5p, let-7f-5p, miR-26b-5p, let-7c-5p, miR-125a-5p, miR-195-5p, miR-23b-3p and miR-374a-5p were determined by RT-qPCR in HEC1A, HEC1B, HEC50 and Ishikawa cells in comparison with the FTE188 control cells are presented (Mean ± SEM; n = 3).

In view of the critical oncogenic responses regulated by these miRNAs (Table 1), we sought to validate the array results with the use of endometrial cancer cell lines. This was carried out using a panel of endometrial cancer cell lines consisting of HEC1A, HEC1B, HEC50, Ishikawa, or RL95 cell lines along with fallopian tube derived epithelial cell line, FTE-188, as control. Expression of the candidate miRNAs in these cell lines were monitored by RT-qPCR analysis. As shown in Fig. 2A, the miR-32-5p, miR-96-5p, miR-141-3p, and miR-210 showed increased expression in endometrial cancer cell lines compared to the control cells FTE188 cells. Whereas, the miRNAs with tumor-suppressive effect, namely, miR-23b-3p, miR-26a-5p, miR-26b-5p, let-7f-5p, let-7c-5p, miR125a-5p, miR-374a and miR-195-5p showed a decreased expression (Figure 2B). The expression profiles of the putative oncomiRs as well as the tumor suppressor miRs were quite similar to their expression profile in endometrial cancer tissue thereby validating the results obtained using the FFPE samples from the endometrial cancer patients.

### Expression Analysis of miRNA-target proteins

**Figure 3:**
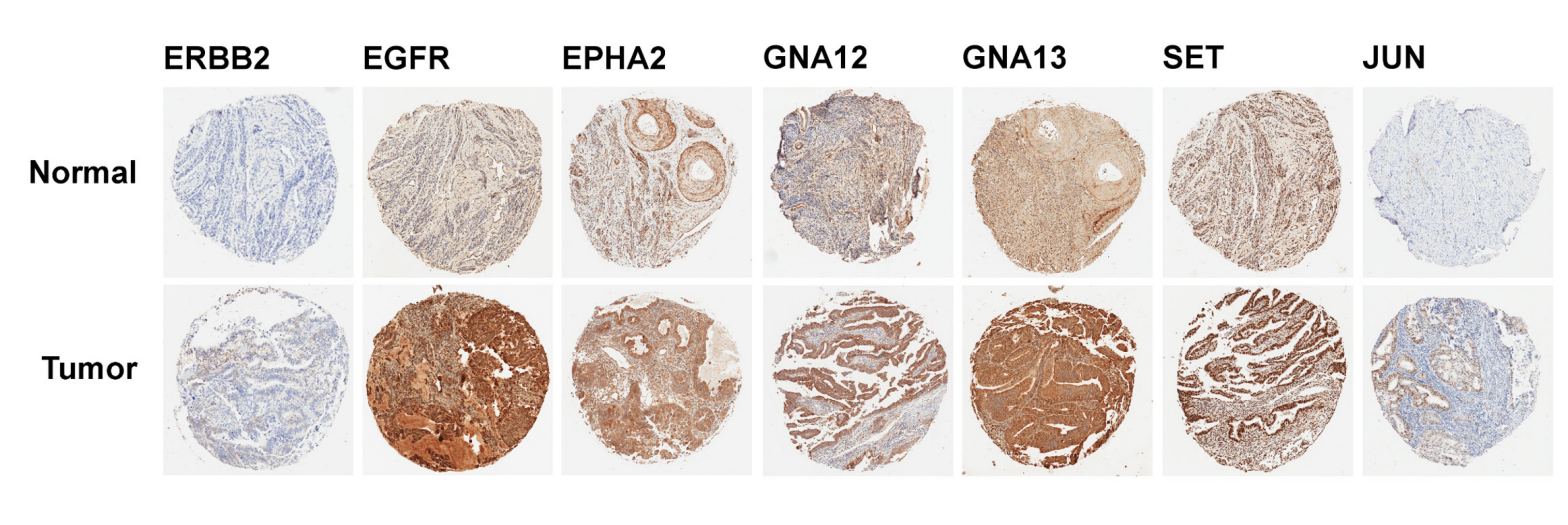
IHC analysis for the expression of target proteins in the endometrial cancer TMA A TMA, constructed from the patient FPPE blocks of 1 mm diameter cores was subjected to IHC analysis using antibodies to specific proteins encoded by the representative target genes for the putative tumor suppressor miRs as detailed in the text. Sample micrographs of the TMA spots developed with 3, 3’-diaminobenzidine (DAB)/HRP staining (brown) for the respective protein are presented; magnification: X 10).

MiRNAs regulate gene expression through RISC-mediated mRNA degradation as well as translational repression [44, 45]. Therefore, we sought to verify the functional relevance of the altered miRNA expressions – especially that of tumor-suppressive miRs – in endometrial cancer pathobiology by monitoring the expression of the target genes in cancer tissue sample. To test whether the decreased expressions of the tumor-suppressive miRs correlate with the increased expression of growth promoting target genes, a tissue micro array (TMA)-based immunohistochemical analysis was carried out. Using the list of downregulated miRNAs identified here, TarBase v7.0 database (http://www.diana.pcbi.upenn.edu/tarbase) was searched for experimentally identified target proteins. A TMA, constructed from the FFPE samples used in this study, was probed with antibodies to specific target gene products. Using this TMA, the expression of *ERBB2, EGFR, EPHA2, GNA12, GNA13, JUN, and SET* messages targeted by miR-26a-5p (*EPHA2*), miR-26b-5p (*EPHA2, GNA13*), let-7f-5p (*GNA13*), miR-23b-3p (*EPHA2, GNA12*), miR-126-3p (*GNA13*), miR-374-5p (*JUN*), and miR-125a-5p (*Jun, ERBB2, SET, EGFR*), were monitored. Our results indicated the increased expression of ERBB2, EGFR, *EPHA2*, GNA12, GNA13, JUN, and SET proteins in cancer tissue samples to endometrial cancer patients compared to normal endometrial tissue (Figure 3). This correlated well with the decreased expressions of the miRNAs that could target the mRNAs that encode these proteins. Although the expression of a few of these proteins such as EGFR, EphA2, ERBB2, and c-Jun have been correlated previously with poor prognosis in endometrial cancer [46-49], the mechanism involved in the upregulation of these oncogenic proteins have not been fully understood. In this context, our observation that the miRNAs that regulate the levels and the activity of mRNAs, and subsequently the levels of the proteins that are encoded by them is highly significant in that it provides the mechanism by which these genes are regulated. Although the specific roles of *GNA12*, *GNA13*, and *SET* genes in endometrial cancer are not known at present, these genes have been identified as oncogenes that do promote tumorigenesis, tumor progression, and metastasis in many different cancers. Thus, our results identify for the first time a potential role for the tumor suppressor miRs and their targeted effect on specific oncogenic proteins in endometrial cancer.

### Therapy response specific miRNA signature

**Figure 4:**
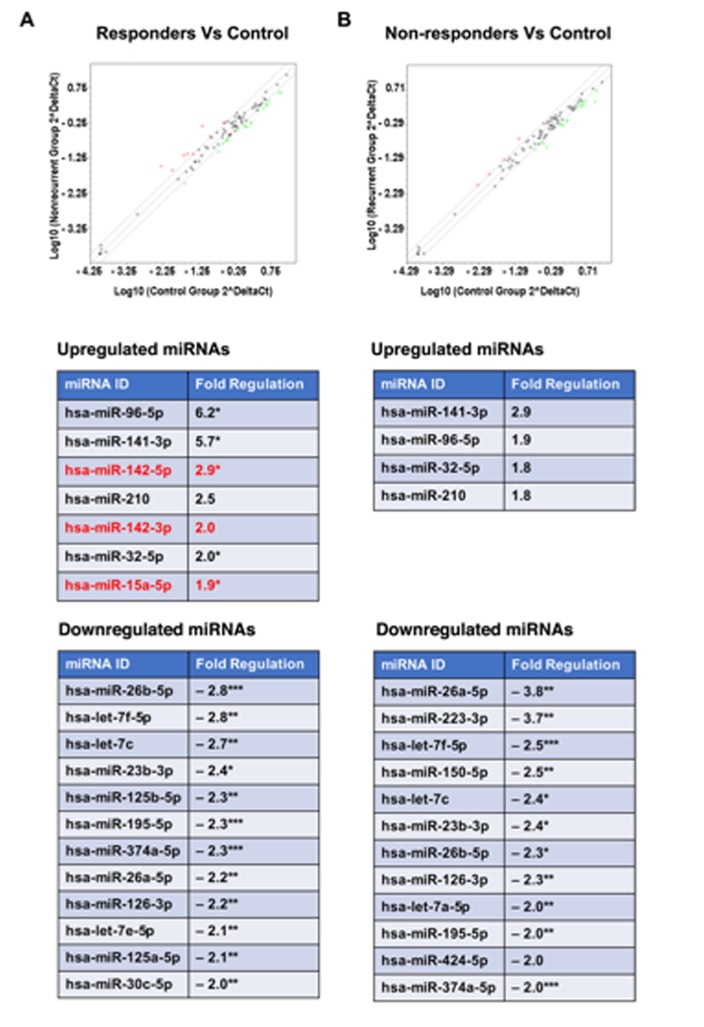
Expression analysis of miRNAs in FFPE samples from different therapy response groups MiRNAs expression profiles in FFPE samples derived from endometrial cancer patients grouped as “non-recurrent/responder” (PFS > 21 months) and recurrent/non-responder (PFS < 21 months) were carried out using Human miFinder miScript miRNA PCR array kit by RT-qPCR. Red open circles indicate each miRNA expression, upregulated miRNA (> 2-fold) as red and downregulated miRNA (<-1.8-fold) as green open circles. A. Scatterplot (Upper Panel) represents results from miRNA profiles showing differentially expressed miRNAs in the endometrial cancer tissue derived from the “responder” group compared to normal tissue. List of miRNAs and the fold changes are presented in the lower panels. B. Scatter plot in the upper panel shows the differential expression of miRNAs between the endometrial cancer tissue from recurrent/non-responder group and normal tissue. Lower panels list the differentially expressed miRNAs and the associated fold changes. Statistically significant difference in the fold changes are indicated by p-values: * - <0.05; ** - <0.01; *** - <0.001.

In an attempt to infer more information about these miRNAs either as diagnostic or prognostic markers, miRNA profiles were compared between the patients who were stratified as therapy responders (PFS > 21 months) and non-responders (PFS < 21 months). Canceling out the miRNAs that were common to both groups indicated, there was no significant difference between the two groups in the profile of downregulated miRNAs (Figure 4). Although miR-150-5p, miR-223-3p, and miR-424-5p appeared specifically downregulated in “non-responder” group, further analysis of the data indicated the samples from the “responder” group also showed a similar (albeit reduced fold decrease, < 2-fold and > 1.5-fold) downregulation (Figure 4). Likewise, the “unique” downregulated miRNA signatures seen with miR-125a-5p and miR-30c-5p in the “responder” group was due to the 2-fold cutoff imposed on the analysis of the data. In fact, these miRNAs showed 1.8-fold and 1.6-fold decrease in the samples from the “non-responder” group. Thus, there does not seem to be a significant difference in the downregulated miRNAs between both the groups of patients. In contrast, the comparison of upregulated miRNAs indicated that the samples from patients with better prognosis showed the upregulation of miRNA, miR-142-3p, miR-142-5p, and miR-15a-5p (Fig. 4). In contrast, the samples derived from patients with PFS of less than 21 months showed little or no expression of miR-142-3p, miR-142-5p, and miR-15a-5p. It is worth noting here that although both the groups showed an increase in the expression of miR-96-5p, the “responder group” showed a relatively stronger expression profile (6-fold increase versus 1.9-fold increase). Considering the role of miR-96 in inhibiting cell proliferation [50] and enhancing chemsensitivity to DNA-damaging therapeutic such as cisplatin [51], it is possible that the increased expression of miR-96-5p along with the miR-142 cluster and miR-15a predicts good prognosis in endometrial cancer.

In light of the known tumor suppressor and chemo-sensitization roles of miR-142-3p, miR-142-5p, and miR-15a-5p, our results point to their potential role in therapy response and identifies them as predictive biomarkers for therapy response. It has been shown that miR-142-3p acts as a tumor suppressor miR that inhibits tumor progression in breast cancer as well as non-small cell lung carcinoma [52, 53]. Likewise, it has been observed that miR-142-5p inhibits cell growth and induces apoptosis in many different cancer cell lines [18, 54, 55]. Tumor suppressor role of miR-142-3p and miR-142-5p has been further validated by the observation that the expression of both miR-142-3p and miR-142-5p are down regulated in hepatocellular carcinoma [56]. Similarly, miR-15a-5p has been shown to act as a tumor suppressor through its ability to silence the expression of many growth promoting oncogenes including C-Jun [57-59]. In fact, it has been shown that the upregulation of miR-142 improves drug sensitivity in several cancers including acute myelogenous leukemia and non-small cell lung cancer [60, 61]. MiR-15a has also shown to enhance the chemosensitivity of cancer cells to therapeutic agents including cisplatin [62, 63]. Although, such a role for miR-142 and miR-15a has not been established in endometrial cancer, it is possible that the upregulation of these miRNAs enhances the chemosensitivity and contributes to the increased PFS time seen in the “responder” category of patients. Cumulatively, these observations along with our results point to the potential utility of these miRNAs as miRNA replacement therapy for endometrial cancer. In addition to defining a specific set of miRNAs and their target genes that can potentially serve as diagnostic biomarker for endometrial cancer, our studies have identified the tumor-suppressive miR-142 cluster and miR-15a as predictive biomarkers for good prognosis. Further studies should define the target genes and mechanism(s) by which these miRNAs confer clinical advantage to endometrial cancer patients.

## MATERIALS AND METHODS

### Cell lines

Endometrial cancer cell line, HEC1A, (ATCC, Manassa, VA) was cultured in McCoy's 5a medium supplemented with 10% FBS (Norcross, GA), 50 U/mL penicillin, 50 µg/mL streptomycin (Mediatech, Manassas, VA) at 37°C in a 5% CO_2_ incubator. Similarly, HEC1B (ATCC, Manassa, VA) was cultured in Eagle's Minimum Essential Medium supplemented with 10% FBS, and penicillin-streptomycin. HEC50 and Ishikawa (a kind gift from Dr. Kimberly K. Leslie, The University of Iowa) was obtained and cultured in DMEM with the indicated supplements as above. The control cell line, FTE188 was cultured in a mixed media at a ratio of 50:50::MCDB105:M199, supplemented with 10% FBS, Epidermal growth factor (10 ng/ml; Sigma, St. Louis, MO), and 50 U/mL penicillin, 50 µg/mL streptomycin.

### RNA extraction and miRNA Analysis

Total RNA including miRNA was extracted from 4x5 micron FFPE sections using Qiagen RNeasy FFPE kit (Qiagen, Valencia, CA) by following the manufacturer's instructions. cDNA synthesis was carried out using a miScript II RT cDNA synthesis kit (Qiagen, Valencia, CA) and the quality of miRNA and efficiency of cDNA synthesis was checked using a miRNA-QC PCR array (Catalog # MIHS-999Z; Qiagen, Valencia, CA). Real-time quantitative PCR (RT-qPCR) was carried out using the cDNA from the above step using miScript SYBR green PCR kit (Bio-Rad, Carlsbad, CA) in a Bio-Rad CFX96 Real time PCR Detection System. The raw Cq values from this assay were exported and data analyzed using software available at http://pcrdataanalysis.sabiosciences.com/mirna following the manufacturer's instructions. Only the samples that passed quality control (QC) PCR array were used in miScript Human miFinder miScript miRNA PCR array kit (MIHS-001Z; Qiagen, Valencia, CA) that profiles the expression of 84 different miRNAs as listed by the manufacturer (https://www.qiagen.com/ch/shop/pcr/primer-sets/miscript-mirna-pcr-arrays/?catno=MIHS-001Z#geneglobe). They are:

hsa-let-7a-5p, hsa-let-7b-5p, hsa-let-7c-5p, hsa-let-7d-5p, hsa-let-7e-5p, hsa-let-7f-5p, hsa-let-7g-5p, hsa-let-7i-5p, hsa-miR-100-5p, hsa-miR-101-3p, hsa-miR-103a-3p, hsa-miR-106b-5p, hsa-miR-122-5p, hsa-miR-124-3p, hsa-miR-125a-5p, hsa-miR-125b-5p, hsa-miR-126-3p, hsa-miR-128-3p, hsa-miR-130a-3p, hsa-miR-140-3p, hsa-miR-141-3p, hsa-miR-142-5p, hsa-miR-142-3p, hsa-miR-143-3p, hsa-miR-144-3p, hsa-miR-146a-5p, hsa-miR-150-5p, hsa-miR-151a-5p, hsa-miR-155-5p, hsa-miR-15a-5p, hsa-miR-15b-5p, hsa-miR-16-5p, hsa-miR-17-5p, hsa-miR-181a-5p, hsa-miR-181b-5p, hsa-miR-185-5p, hsa-miR-186-5p, hsa-miR-18a-5p, hsa-miR-191-5p, hsa-miR-194-5p, hsa-miR-195-5p, hsa-miR-196b-5p, hsa-miR-19a-3p, hsa-miR-19b-3p, hsa-miR-200c-3p, hsa-miR-20a-5p, hsa-miR-21-5p, hsa-miR-210-3p, hsa-miR-22-3p, hsa-miR-222-3p, hsa-miR-223-3p, hsa-miR-23a-3p, hsa-miR-23b-3p, hsa-miR-24-3p, hsa-miR-25-3p, hsa-miR-26a-5p, hsa-miR-26b-5p, hsa-miR-27a-3p, hsa-miR-27b-3p, hsa-miR-28-5p, hsa-miR-29a-3p, hsa-miR-29b-3p, hsa-miR-29c-3p, hsa-miR-302a-3p, hsa-miR-302b-3p, hsa-miR-302c-3p, hsa-miR-30a-5p, hsa-miR-30b-5p, hsa-miR-30c-5p, hsa-miR-30d-5p, hsa-miR-30e-5p, hsa-miR-32-5p, hsa-miR-320a, hsa-miR-374a-5p, hsa-miR-376c-3p, hsa-miR-423-5p, hsa-miR-424-5p, hsa-miR-425-5p, hsa-miR-7-5p, hsa-miR-9-5p, hsa-miR-92a-3p, hsa-miR-93-5p, hsa-miR-96-5p, hsa-miR-99a-5p

The QC-passed cDNA samples were subjected to RT-qPCR using the miScript SYBR green PCR kit (Bio-Rad, Carlsbad, CA) in a Bio-Rad CFX96 Real time PCR system. The raw Cq values from these assays were exported and data analyzed using software available at http://pcrdataanalysis.sabiosciences.com/mirna following the instructions. The data was normalized to the house keeping genes in the array and miRNAs that were up- or down-regulated by 1.8-fold compared to the control group along with the p-values were extracted from the software.

### Expression of miRNA in cell lines

Endometrial cancer cell lines HEC1A, HEC1B, HEC50, Ishikawa and FTE188 control cells were cultured and collected during their log phase of growth and total RNA including the miRNA was extracted using RNAeasy plus mini kit (Qiagen, Valencia, CA). The RNA was quantitated using a nanodrop and cDNA synthesis was carried out as described above. RT-qPCR analysis was carried out using the miScript Primer Assays (Qiagen, Valencia, CA) specific for the miRNA of interest. The data were analyzed in CFX Manager software and exported to GraphPad Prism (La Jolla, CA) where graphs and statistical analyses were done.

### Immunohistochemical staining of tissue microarray (TMA)

TMA was constructed at the Stephenson Cancer Center's tissue pathology core using Veridiam VTA-100 Tissue Arrayer [7]. IHC-staining of the TMA with the antibodies of interest was carried out at the SCC tissue pathology core that utilizes automated Leica Bond III for IHC staining. In brief, FFPE tissues were sectioned at desired thickness (4µm) and mounted on positively charged slides. The slides were dried overnight at room temperature and incubated at 60°C for 45 minutes followed by deparaffinization and rehydration in an automated Multistainer (Leica ST5020). Subsequently, these slides were transferred to the Leica Bond-III^TM^, treated for target retrieval at 100°C for 20 minutes in a retrieval solution, either at pH 6.0 or pH 9.0. Endogenous peroxidase was blocked using peroxidase-blocking reagent, followed by the selected primary antibody incubation for 60 minutes. For the secondary antibody, post-primary IgG-linker and/or Poly-HRP IgG reagents was used. 3, 3′-diaminobenzidine tetrahydrochloride (DAB) was used as the chromogen and the slides were counterstained with hematoxylin. Completed slides were dehydrated (Leica ST5020), and mounted (Leica MM24). Antibody specific positive control and negative control (omission of primary antibody) were parallel-stained.

### Scanning and analysis of IHC slides

The IHC stained TMA slides were scanned into an Aperio slide scanner. The TMA are segmented and individual spots assigned to respective patients using a TMA lab software which works in conjunction with the spectrum software from Leica Aperio that analyzes the intensity of the staining of various proteins.

### Data analysis

The raw Cq values from the miRNA assays were exported from the Bio-Rad CFX96 real-time PCR system (Carlsbad, CA) and data analyzed using the web-interface software available at http://pcrdataanalysis.sabiosciences.com/mirna following the manufacturer's instructions. Briefly, the data was normalized to the house keeping genes built within each array. Scatterplots of the miRNAs that were up- or down-regulated by 1.8-fold compared to the control group along with the p-values were calculated using the web-interface software. The heatmap for miRNAs correlating the patient samples was generated using Matrix2png, a utility for visualization of data matrix (http://www.chibi.ubc.ca/matrix2png/). All statistical analyses were carried out using GraphPad Prism (La Jolla, CA) by two-tailed student's t-test.
